# Influence of Cuticle Nanostructuring on the Wetting Behaviour/States on Cicada Wings

**DOI:** 10.1371/journal.pone.0035056

**Published:** 2012-04-20

**Authors:** Mingxia Sun, Aiping Liang, Gregory S. Watson, Jolanta A. Watson, Yongmei Zheng, Jie Ju, Lei Jiang

**Affiliations:** 1 Key Laboratory of the Zoological Systematics and Evolution, Institute of Zoology, Chinese Academy of Sciences, Beijing, China; 2 School of Pharmacy and Molecular Sciences, James Cook University, Townsville, Queensland, Australia; 3 Key Laboratory of Bio-Inspired Smart Interfacial Science and Technology of Ministry of Education, School of Chemistry and Environment, Beihang University, Beijing, China; 4 Center of Molecular Sciences, Institute of Chemistry, Chinese Academy of Sciences, Beijing, China; RMIT University, Australia

## Abstract

The nanoscale protrusions of different morphologies on wing surfaces of four cicada species were examined under an environmental scanning electron microscope (ESEM). The water contact angles (CAs) of the wing surfaces were measured along with droplet adhesion values using a high-sensitivity microelectromechanical balance system. The water CA and adhesive force measurements obtained were found to relate to the nanostructuring differences of the four species. The adhesive forces in combination with the Cassie-Baxter and Wenzel approximations were used to predict wetting states of the insect wing cuticles. The more disordered and inhomogeneous surface of the species *Leptopsalta bifuscata* demonstrated a Wenzel type wetting state or an intermediate state of spreading and imbibition with a CA of 81.3° and high adhesive force of 149.5 µN. Three other species (*Cryptotympana atrata, Meimuna opalifer* and *Aola bindusara*) exhibited nanostructuring of the form of conically shaped protrusions, which were spherically capped. These surfaces presented a range of high adhesional values; however, the CAs were highly hydrophobic (*C. atrata* and *A. bindusara*) and in some cases close to superhydrophobic (*M. opalifer*). The wetting states of *A. bindusara*, *C. atrata* and *M. opalifer* (based on adhesion and CAs) are most likely represented by the transitional region between the Cassie-Baxter and Wenzel approximations to varying degrees.

## Introduction

Research on the superhydrophobic and self-cleaning properties of natural materials, such as the famous lotus leaf, has been undertaken for more than ten years [1], [2]. The original impetus for studies in this area stems from the low adhesion observed between lotus leaves and water or other contaminants [1], [3]. It has been shown, however, that the wetting properties on different regions of the leaf vary. For instance the flat folds around the margin of the lotus leaf have a much larger contact angle (CA) hysteresis (CAH) than that of the upper surface of the lotus leaf including the micro-papillae [4]. There are a number of other natural superhydrophobic surfaces which also demosntsrate a large CAH. The rose petal surface, for example, consists of hierarchical micropapillae and nanofolds, which provide a sufficient roughness for superhydrophobicity and yet at the same time a high adhesive force with water [5]. In this case the pitch values of microstructures and density of nanostructures play an important role [6]. For scallions and garlic, hydrophobic defects result in contact line pinning and high CAH [7].

In general, a range of factors influence the adhesive properties of solid surfaces such as chemical compositions, density [8], real area of contact, surface energy effects [9], surface roughness [10] and the apex geometry [11]. A contributing aspect for the resulting high adhesion between some surfaces and water (besides the capillary force [12] and the negative pressure produced by the volumes of sealed air [11]) has been ascribed to van der Waals forces [11]–[13].

For some biological samples, the heterogeneous nature of the surface also plays an important role in adhesive properties [6]. Many naturally occurring nano-structures have demonstrated functional efficiencies which are superior to man-made technologies. One of the most noteworthy nano-composite materials is the insect cuticle [14]. Recently micro- and nano-structures found on insect cuticle have been shown to exhibit a range of impressive and remarkable properties such as superhydrophobicity, directed wetting, self cleaning and ultra-low adhesion [15]–[26]. The cuticle on the wings of insects demonstrates a wide variety of small scale structuring. The cicada wing is a prime example. A range of interesting properties have been demonstrated on the surfaces of cicada wings with functions and functional efficiencies related to the structure parameters (shape, size, spacing etc). Current studies have focused on a number of aspects such as wettability, antireflection, self cleaning, particle adhesion, antimicrobical activity, cell growth platforms,, material properties and biomimetic fabrication of the nanostructures [27]–[38]. Indeed some of these atributes (self-cleaning, antimicrobical, cell growth, antirelfection) suggest that biomimetic fabrication may potentially have a wide variety of applications ranging from clinical biomaterials (e.g., ocular tissue engineering strategies), antibacterial surfaces/implants and self-cleaning medical based surfaces [37], [38]. We have recently reported on the interaction of water with cicada forewings [39]. We showed significantly different wettabilities associated with distinct differences in surface patterning of nanostructures. The primary focus of this study is an investigation of the wetting and adhesive properties of the wing membranes of four specific cicada species.The study examines the adhesional dependence with the different membrane morphologies and analyzes the mechanism of high adhesion through modeling to ascertain the most likely wetting states.

## Results and Discussion

### Surface morphology and wettability

**Figure 1:**
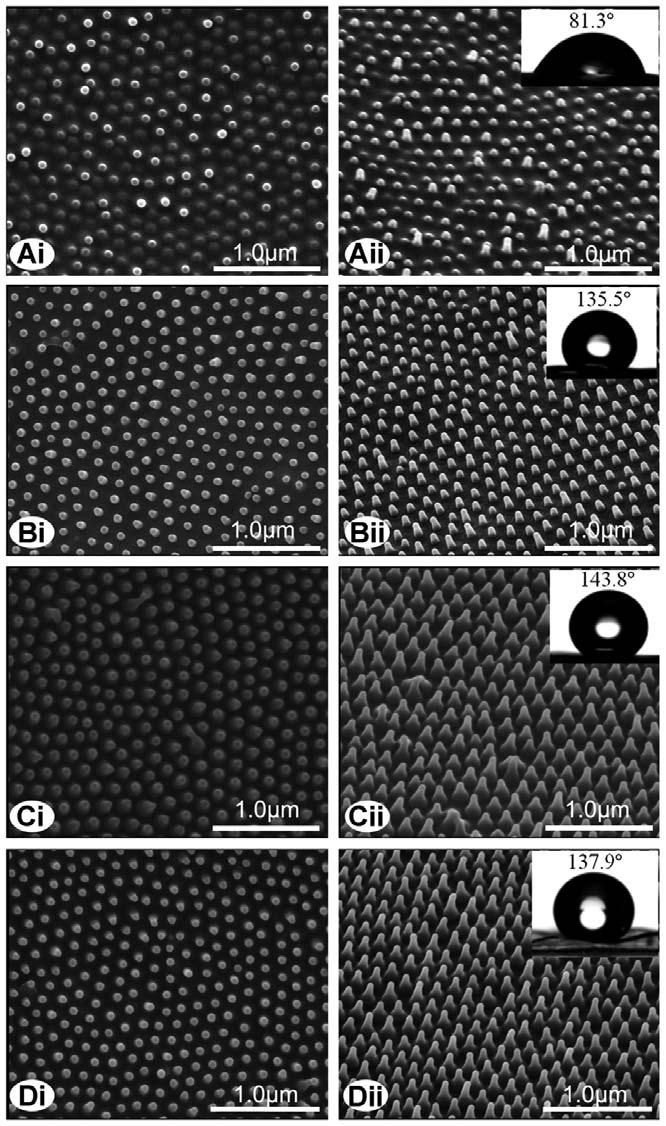
SEM images of four species of cicada wing surfaces. (A) *Leptopsalta bifuscata*; (B) *Aola bindusara*; (C) *Meimuna opalifer*; (D) *Cryptotympana atrata*. The insets are the optical images of water droplets on the wing surfaces. Subsections (i) show a top view and (ii) show a 30° inclined view of the wing membranes.

Unlike the hierarchical micro- and nanostructuring of the lotus leaf and rose petal, the cicada wing surfaces exhibited a single level of roughness where small nanoscale structures were observed. Fig. 1 shows the fine protrusions and different morphologies found on the four species of cicada wing surfaces studied. The species *Leptopsalta bifuscata* has nanostructuring comprising of a dome shape resulting in spherically capped surface structures (Fig. 1A). Some of the taller structures exhibit a cylindrical shape which is spherically capped. This type of surface represents the most disordered/inhomegeous of the structuring in this study. The relevant structure parameters are: average basal diameter (*d*) of 90 nm, basal spacing (*s*) of 117 nm and height (*h*) of 200 nm. On this type of surface the wettability is exhibited as hydrophilic with a low CA of 81.3° (inset in Fig. 1Aii).

The other three cicada species demonstrate a more homogeneous surface topography with conical shaped nanostructures and display a stronger hydrophobicity than the cylindrically shaped structuring of *L. bifuscata* (Fig. 1, Table 1). The cicada species *Aola bindusara* (Fig. 1B) clearly shows a more ordered structuring than *L. bifuscata* with a more consistent structure height (lower SD) and a higher CA of 135.5°. The structuring, although more conical in shape, could still be approximated as spherical at the apex of structures. The spherical apex appears swollen on most of the nano-structures. The other two cicada species (*Meimuna opalifer* with a CA of 143.8° and *Cryptotympana atrata* with CAs of 132.7° or 137.9°) exhibit more conically shaped protrusions as seen in Figs. 1C and 1D, respectively. These cicadas also demonstrate a much higher structure height (*h*) on the wing surface compared to *A. bindusara* (up to twice the height, see Fig. 1 and Table 1). Structures on *M. opalifer* exhibit significantly larger *d* values (ca. 148 nm) and smaller *s* values (ca. 48 nm) when compared to the other species under investigation. So, the slightly swollen apex and the significantly larger basal diameter and height coupled with the small spacing of protrusions appears to efficiently increase hydrophobicity of the regularly patterned cicada surfaces. The swollen apex shape may be an attempt to reduce weight and material.

**Table 1:** Mean values and standard deviations (in brackets) of nanostructure parameters including diameter (*d*), spacing (*s*), height (*h*) and the density of protrusions, the adhesive force, measured contact angles (CAs) and calculated contact angle hysteresis (CAH), *θ*_w_ and *θ*_c_ based on the Wenzel and Cassie-Baxter wetting states on dry wing surfaces of four species of cicadas collected on different dates.

Species	Collection dates	*d*	*s*	*h*	Density	Force	Measured	Calculated		
	D.M.Y	nm	nm	nm	1/10000 µm^2^	µN	CAs°	CAH°	*θ*_w_°	*θ*_c_°
*L. bifuscata*	22.8.1964	90(5)	117(13)	200(52)	30	149.5	81.3(8.3)	9.9	133.9	149.3
*A. bindusara*	29.6.1956	84(4)	91(13)	234(18)	42	123.0	135.5(5.2)	1.0	157.4	146.0
*M. opalifer*	23.7.1998	148(6)	48(5)	418(38)	33	131.3	143.8(6.0)	3.3	-	125.2
*C. atrata*	25.8.2010	85(5)	90(8)	462(34)	42	170.0	137.9(1.9)	1.5	-	145.6
	11.8.1951	95(5)	90(8)	410(49)	37	171.9	32.7(4.0)	2.5	-	143.5

**Footnote:** -Values fall outside the boundaries of the Wenzel Equation. Height values were calculated from inclination of the surface by 30°.

Protrusion height differences were also observed between *C. atrata* samples collected in 2010 and 1951. The dry sample collected in 2010 showed a height of 462 nm compared to the dry sample (ca. 410 nm) collected in 1951 (Table 1). This difference may be due to the hydration/age effects (weathering and/or removal of the outermost layering). The differences in the structural parameters for samples of this age can also be representative of variation within the species or indicate a sub-species.

When compared to the other species (including the older, dry samples of the same species), the fresh wing surface of the cicada *C. atrata* showed the strongest hydrophobicity with a CA value of 147.4±6.4° (Fig. S1) approaching superhydrophobic values. In contrast, the naturally dried samples had a measured CA of ca. 137.9° (Table 1). This phenomenon is possibly due to changes in the chemical constituents existing on the wing surfaces. The wing surface membrane consists of a wax layer [39], and this wax layer may degrade over time yielding a more hydrophilic chemistry. Alternatively, the decreased structure height may also contribute to the lower CA, although this effect is expected to contribute less as seen by comparison of the CAs on dry samples of *M. opalifer* and *C. atrata* (both these samples have similar nano structure dimensions). These observed differences (fresh and dried samples) will be explored in future studies.

### Surface CA hysteresis and adhesive force

**Figure 2:**
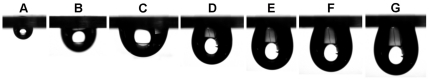
Optical images of different volumes of water droplets hanging on the dry wing surface of cicada *Cryptotympana atrata* collected in the year 2010. (A) 3 µL; (B) 20 µL; (C) 30 µL; (D) 35 µL; (E) 37 µL; (F) 39 µL; (G) 42 µL.

The wing surfaces of the four species of cicadas studied showed different wettabilities. Water droplets of 3 µL stayed pinned to all wing surfaces, including fresh and dry samples, even when they were inverted (Fig. S2). This demonstrates that the weight of the water droplet is small in comparison to the surface adhesional force. When the volume of the droplet was increased to 10 µL, the droplet on the surface of *A. bindusara* fell when tilted to ca. 65°, with the weight sufficient to overcome a weak pinning of the contact line on the corner of the protrusions. Interestingly, the CAH measurements were varied on the cicada samples (see Table 1) with the highest value recorded on *L. bifuscata* suggesting that the surface may exhibit one of the highest adhesive forces. The maximum volume of a water droplet which remained hanging was 42 µL on the dry sample of *C. atrata* collected in the year 2010 (Fig. 2). This highlights that the extent of the adhesive force between water and the cicada wing surface can be quite large.

**Figure 3:**
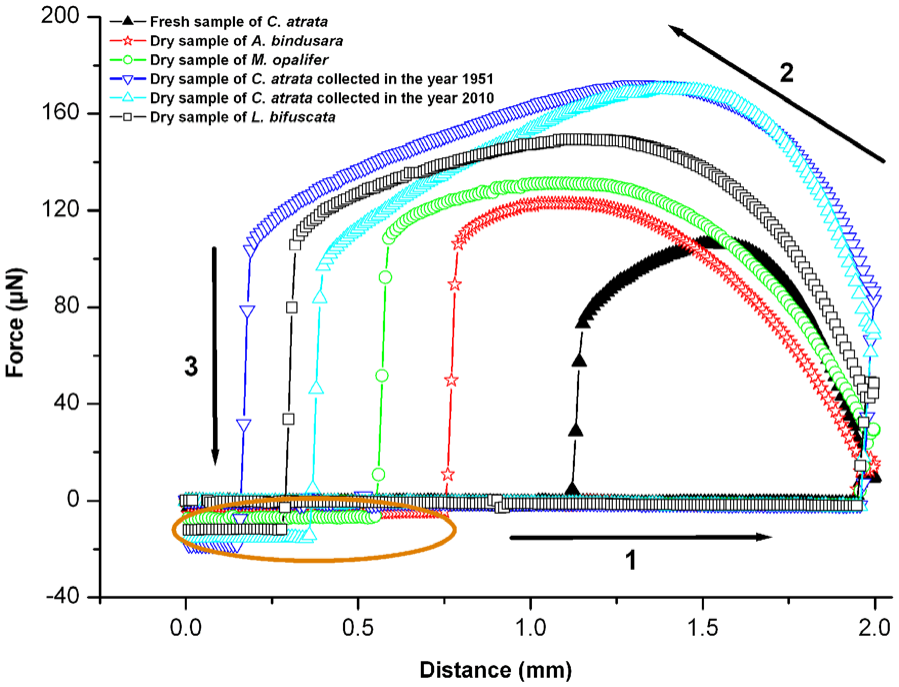
Force-distance curves recorded before and after the water droplet makes contact with the cicada wing. Process 1: the cicada wing surface approaches the water droplet. Process 2: the cicada wing surface leaves the water droplet after contact. Process 3: the cicada wing surface breaks away from the water droplet. The negative values in the circled section indicate an amount of water still remaining on the wing surfaces when separating from each other.

To determine the magnitude and differences of adhesion among these cicada wing surfaces the adhesive force was measured using a high-sensitivity microelectromechanical balance system. The method utilized in this study to assess the adhesive force between a solid surface and water can be also used to analyse a number of other solid/liquid interfacial interactions [10]. As shown in Fig. 3, the fresh sample of *C. atrata* resulted in the lowest adhesive force of only 106.3 µN. This is in contrast to the dried samples which exhibited the largest adhesive forces recorded, ca. 170.0 µN and 171.9 µN for the samples collected in 2010 and 1951, respectively (Table 1). The dried samples of *C. atrata* also displayed similar wetting properties with CAs of 137.9° and 132.7°, respectively, despite the different collection dates. Thus changes in wetting properties appear to occur rapidly in the first 3 months, with no significant differences beyond this. The CAH was suprisingly low on the *C atrata* membranes considering the larger magnitude of the adhesion forces in comparision to the other species. Thus a more anisotropic force relationship in relation to water droplet movement appears to be present on this cicada surface. This suggests that the contact area of water with the surface is large enough to promote high adhesion whilst allowing lateral movement to be less hindered. This may potentially be due to the high structure density combined with the different structure morphology (high conically shaped protrusions yeilding large contact area/large adhesion with water droplets) in comparision to the other ciacda structures. The gentle tapering of the structures may be one of the contributing factors which promotes low CAH values.

Among all the dry samples, the surface of *A. bindusara* (Fig. 1B) showed a minimum adhesion value of the order of 123.0 µN (Table 1). It also showed the lowest CAH of all the samples. These lower values can explain why a water droplet of 10 µL cannot remain pinned to the surface when the wing membrane is inverted. The hydrophilic surface of *L. bifuscata* (CA of 81.3°) (Fig. 1A) yielded an adhesive force of 149.5 µN. This value is greater than that of *M. opalifer* (131.3 µN) which is the most hydrophobic dry surface with a CA of 143.8°. The CAH value measured on *M. opalifer* was lower (3.3°) than that of *L. bifuscata* (9.9°) and is conducive to the higher adhesion measured on the later surface.

High adhesion on the rose petal, has been attributed to low height and structure density [6]. Interestingly, the cicada with the higher structure height (*C. atrata* ca. 462 nm) showed a higher adhesional value when compared to *A. bindusara* which also has the same structure density (42×1/10000 µm^2^). However, the surface of *L. bifuscata* which had the lowest protusion density (30×1/10000 µm^2^) but a similar protusion height to *A. bindusara*, resulted in a larger adhesion value (Table 1). This observation is most probably due to the more disordered and inhomogeneous landscape of the *L. bifuscata* cuticle. It appears that the height and density are not the only determining factors for the adhesional contact. The overall morphology and degree of order may also play an important role.

As indicated by the circled region in Fig. 3, as the water droplet breaks away during process 3, the negative values of adhesive forces on the vertical axis indicate that a small amount of water remains pinned on the wing during the retraction cycle. The larger the negative value, the greater the amount of water which remained adhered to the wing. The smallest adhesion value (106.3 µN) on the fresh sample of *C. atrata*, indicated that very little water remained (no negative retract forces in Fig. 3). The dry sample of *C. atrata* collected in the year 1951 however, revealed the greatest amount of water was retained on the wing membrane (i.e., the largest negative retract force value). This surface also displayed the largest measured adhesion of 171.9 µN.

### Wetting states based on water interactions

From the viewpoint of force and energy dissipation, when contacting with a solid, the surface energy of a drop is gradually lowered until ultimately reaching an equilibrium position [40]. On contacting the protrusions, water droplets tend to lower the center of the mass of the liquid, which surface tension opposes. At this moment, the downward gravitational force of the water droplet exceeding the surface tension allows it to move along the wall of protrusions. Different contact areas of water and protrusions result in different interaction forces. The larger the force exerted on the protrusions, the greater the accumulative energy consumed by the reaction force produced by the protrusions. Consequently, if it becomes more diffcult for water to sink further into the structuring, the contact angle will increase.

**Figure 4:**
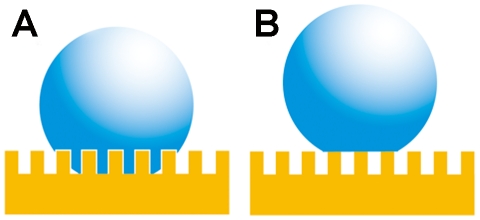
Two wetting states of a liquid on the rough solid surface. (A) Wenzel model; (B) Cassie-Baxter model.

A number of theories purport to describe the effect of surface roughness on hydrophobicity. As many as six wetting states among two different substances, such as solid-liquid and/or biological surfaces, have been reported [5], [41]. In order to attempt to understand the wetting states of the cicada wing surfaces and the different adhesive forces, we have calculated the predicted contact angles using the Wenzel and Cassie-Baxter models. These two regimes represent two extreme states. The Wenzel model [42] makes the assumption that, when a liquid drop is placed on a surface consisting of protrusions, the liquid will fill the open spaces, as shown in Fig. 4A. This model predicts that roughness of the surface reinforces both hydrophobicity and hydrophilicity. Cassie and Baxter [43] on the other hand, consider the microstructures to be a heterogeneous surface composed of solid and air (Fig. 4B). The crucial assumption is that the space between asperities will remain filled with air with the droplet sitting at the very top of the surface features.

In the Wenzel state (impregnating wetting regime) the CAH is large, while in the Cassie-Baxter state CAH will be low. Low CAH also implies low adhesion [6]. The Wenzel and Cassie-Baxter models describe static droplets at equilibrium and allow calculation of the contact angle for the two conditions [42], [43],
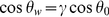
(1)

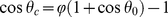
(2)where 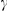 is the roughness factor (the ratio of actual area to geometry projected area of surface), 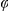 is solid fraction in contact with the liquid, 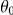 is the contact angle on a smooth surface of the same material, 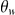 and 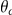 are the apparent contact angles on a rough surface. Thus, the roughness factor (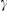) of wing surfaces was calculated using the following equation (3):
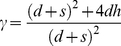
(3)and the fraction (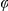) was determined using equation (4):
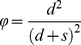
(4)where 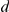, 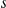 and 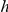 are the diameter, spacing and height of protrusions, respectively. Given 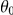 = 105° [15], and substituting 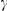 and 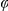 into the two model equations, the values of 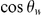 and 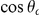 were obtained (Table S1). In Table 1, the density of protrusions is defined as the average number of protrusions in the area of 100×100 µm^2^.

**Figure 5:**
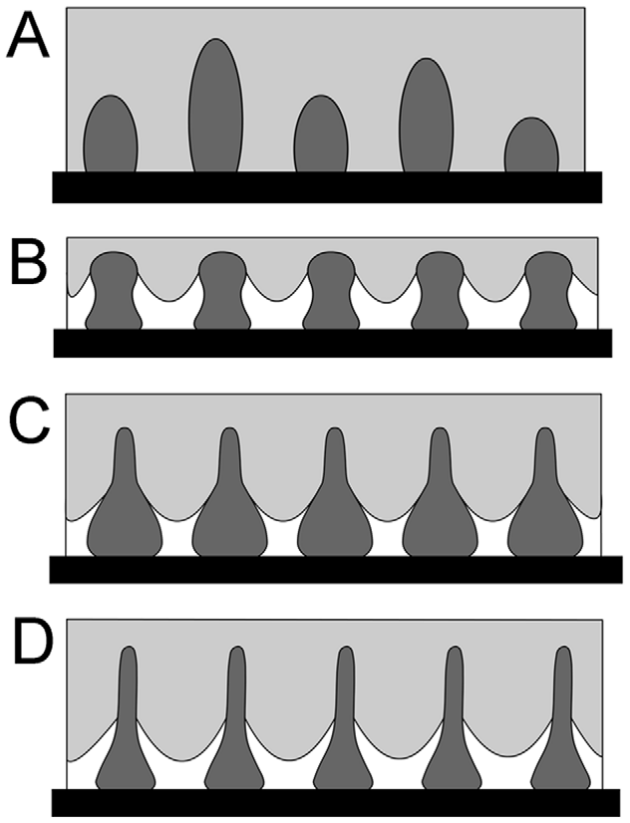
Schematic illustrations of a droplet of water in contact with the cicada wing surface. (A) *Leptopsalta bifuscata*; (B) *Aola bindusara*; (C) *Meimuna opalifer*; (D) *Cryptotympana atrata*. The dark grey areas represent protrusions, the light grey areas show the water droplet remaining on the surfaces and the black lines show the interface of solid, liquid and gas.

Of the cicada wing surfaces studied here, *L. Bifuscata* demonstrated the lowest hydrophobicity with a measured CA of 81.3°. This value is much lower than the one predicted by both the Wenzel (133.9°) and Cassie-Baxter states (149.3°) (Table 1). The contact conditions (based on high adhesion and calculated CA) are more closely aligned with the Wenzel approximation (Fig. 5A), however this may only reflect the wetting state directly beneath the water droplet. The measured low CA may also be a consequence of a thin water film developing in the cuticle nano-texture where the drop sits upon a mixture of solid and liquid. The penetration front spreading beyond the drop is most likely a consequence of the disordered and inhomogeneous surface patterning of the cuticle. Thus the patterning may contribute to this equilibrium wetting state, i.e., the intermediate state of spreading and imbibition due to hemi-wicking [44].

A transitional state between Wenzel and Cassie-Baxter states could be expected on the other species from the measured and predicted CAs and adhesional values (see Table 1). The high contact angle oberved on *A. bindusara* combined with the lower adhesion value suggests that the surface structuring is more likely to be in the transitional region nearing the Cassie-Baxter state. The measured contact angle of 135.5° is closer to the predicted value of 146.0° from the Cassie-Baxter model (Table 1). The slightly swollen tops of protrusions may aid in preventing the water droplets from contacting the underlying membrane, keeping the contact line high on the protrusions (Fig. 5B). Thus an energy hurdle is required to overcome the geometrical barrier presented to the solid-liquid contact line.

The larger diameter, smaller spacing (at the base) and greater height of protrusions on the surface of *M. opalifer*, achieved a near superhydrophobic surface. The high CA and moderate adhesion value indicates surface wetting is most likely to be in the transitional region between the Cassie-Baxter and Wenzel states (Fig. 5C). The hydrophobicity (CA) of this sample and *C. atrata* were similar, however the adhesion values on *C. atrata* were significantly higher (over 20%). The patterning on both samples showed similar structure height, however the density of structures was higher on *C. atrata* (Table 1). So it is likely that the increased contact area (and thus enhancement of the van der Waals' forces) between the cicada wing surface and water leads to the high adhesion. The long slender nature of the protrusions on these samples would require significant energy for the water to invade completely to the bottom of the protrusions. The measured CAs for *C. atrata* are lower than the calculated angles (Table 1) based on Cassie-Baxter predictions (and cos*θ_w_*<−1) (Table S1), so this cuticle is also likely to be in the transitional region (Fig. 5D).

### Conclusions

We have explored the relationship between structure, wettability and adhesion of several cicada wings. Homogeneity of protrusion patterning on the samples strengthened the hydrophobic properties of the surfaces. As well protusion shape (e.g., nanostructrues with a swollen apex) and density of nanostructuring also play a role in determining adhesion and hydrophobicity. It is also likely that the enhancement of van der Waals forces between water and cicada wings (as a consequence of increased surface wetting) result in a greater force of adhesion. The schematic wetting models of surface nanostructures explain why cicada wings may be highly hydrophobic but not superhydrophobic.

Cicada wing surfaces have been shown in previous studies to exhibit interesting features such as high Young's modulus values (3.7 GPa [27]) and heat resistance (200°C [33]), and have a broad range of applications (e.g. bio-templating) [33]–[35]. We have shown that slight changes in morphologies can achieve the purpose of tunable wettability and adhesion. It is expected that a surface with a sufficiently high adhesive force to a liquid will have many potential applications, such as in liquid transportation and capture without loss and in the analysis of very small volumes of liquid samples.

## Materials and Methods

### Ethics Statement

No specific permits were required for the described field studies. The insect species collected are not endangered or protected.

### Preparation of samples

Four different species of cicadas were investigated in this study: *Cryptotympana atrata* (fresh and dry samples), *Meimuna opalifer* (dry sample), *Aola bindusara* (dry sample) and *Leptopsalta bifuscata* (dry sample) collected in Beijing, Shaanxi, Yunnan and Hebei of China, respectively, on different dates (Table 1). The fresh forewing of *C. atrata* was used immediately after collection in order to study age effects on the microstructure, wetting characterization and adhesive forces. The experiments were duplicated on the forewing of *C. atrata* (after naturally drying for more than three months) and the other three species. The apical 1/3 of the forewing of each cicada species was excised with a scalpel, rinsed using deionized water to remove environmental contaminants and then dried at room temperature for measurements on the remigium region.

### Microstructure observation

The cicada forewings were attached onto a platform using conductive adhesive. The nanostructures were observed and evaluated on an environmental scanning electron microscope (ESEM) (Quanta 200 FEG, FEI, Eindhoven, Netherlands) after being coated with a thin layer of gold. The wings were titled to 0° and 30°, respectively, for the three dimensional observations. The calculated structure parameters, including basal diameter (*d*), basal spacing (*s*) and height (*h*) of protrusions on the surfaces were an average value of twenty measurements, and their standard deviations (in brackets) were calculated (Table 1).

### Measurements of CA, sliding angle (SA) and CAH

Static CAs and SAs were measured on a Data-Physics OCA 20 contact angle system (Filderstadt, Germany) at ambient temperature using the sessile drop method. The wings were fixed on glass slides with double-sided adhesive. 3 µL water droplets were used for CA measurements and 10 µL water droplets added to wing surfaces via a syringe were used for SA measurements. CAH is defined as the difference between the advancing and receding angles, which were recorded by adding or removing a small amount of water from the drop. Each wetting angle was measured at five to ten different points on each wing, and average values and standard deviations (in brackets) were calculated (Table 1).

### Measurements of adhesive force

The force required to retract the water droplet away from the cicada wing was measured using a high-sensitivity microelectromechanical balance system (Data-Physics DCAT 11, Germany). A 5 µL water droplet was suspended with a metal ring in the first instance, and the cicada wing was placed on the balance table. The cicada wing was moved upward at a constant speed of 0.05 mm s^−1^ until contact with the water droplet. The force increased gradually until it reached its maximum, and the shape of the water droplet changed from spherical to elliptical. When the cicada wing was moved down, the contact force sharply reduced to zero and the shape of the water droplet reverted back to spherical. At this time the maximum force is the adhesion force of water with cicada wing.

## Supporting Information

Figure S1 The optical image of water droplet on the fresh sample of wing surface of cicada *Cryptotympana atrata* collected in the year 2010.
(TIF)

Figure S2 Optical images of water droplets hanging into four species of cicadas. (A) *Leptopsalta bifuscata*; (B) *Aola bindusara*; (C) *Meimuna opalifer*; (D) *Cryptotympana atrata* collected in the year 1951. The volume of water droplet is 3 µL in left column and 10 µL in right column.
(TIF)

Table S1 Mean values and standard deviations (in brackets) of nanostructure parameters including diameter (*d*), spacing (*s*) and height (*h*) of protrusions, roughness factor (*r*), solid fraction (*ϕ*) in contact with the liquid, cos*θ_w_* and cos*θ_c_* based on the Wenzel and Cassie-Baxter wetting states on dry wing surfaces of four species of cicadas.
(DOC)
