# Supplementary figures and images for: Influence of Cuticle Nanostructuring on the Wetting Behaviour/States on Cicada Wings
Source: PLoS One. 2012 Apr 20;7(4):e35056. doi: 10.1371/journal.pone.0035056 (PMC3335046; doi:10.1371/journal.pone.0035056)

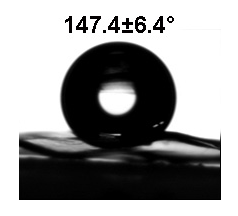

Supplement: Figure S1 — The optical image of water droplet on the fresh sample of wing surface of cicada Cryptotympana atrata collected in the year 2010. (TIF) [file pone.0035056.s001.tif]

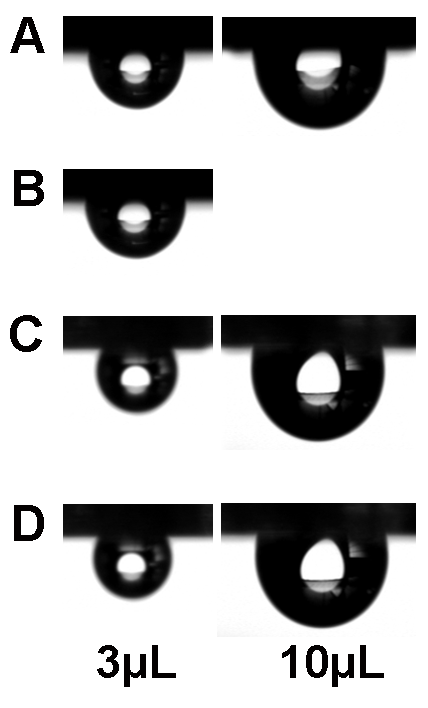

Supplement: Figure S2 — Optical images of water droplets hanging into four species of cicadas. (A) Leptopsalta bifuscata; (B) Aola bindusara; (C) Meimuna opalifer; (D) Cryptotympana atrata collected in the year 1951. The volume of water droplet is 3 µL in left column and 10 µL in right column. (TIF) [file pone.0035056.s002.tif]
