# Supplementary material for: Influence of Cuticle Nanostructuring on the Wetting Behaviour/States on Cicada Wings
Source: PLoS One. 2012 Apr 20;7(4):e35056. doi: 10.1371/journal.pone.0035056 (PMC3335046; doi:10.1371/journal.pone.0035056)
Supplement: Table S1 — Mean values and standard deviations (in brackets) of nanostructure parameters including diameter (d), spacing (s) and height (h) of protrusions, roughness factor (r), solid fraction (ϕ) in contact with the liquid, cosθw and cosθc based on the Wenzel and Cassie-Baxter wetting states on dry wing surfaces of four species of cicadas. (DOC) [file pone.0035056.s003.doc]

**Supporting Information**

**Table S1.** Mean values and standard deviations (in brackets) of nanostructure parameters including diameter (*d*), spacing (*s*) and height (*h*) of protrusions, roughness factor (*r*), solid fraction (*ϕ*) in contact with the liquid, cos*θ_w_* and cos*θ_c_* based on the Wenzel and Cassie-Baxter wetting states on dry wing surfaces of four species of cicadas.

| Species | *d* | *s* | *h* | Wenzel state | | Cassie-Baxter state | |
| --- | --- | --- | --- | --- | --- | --- | --- |
|  | nm | nm | nm | *r* | cos*θ*_w_ | *ϕ* | cos*θ*_c_ |
| *L. bifuscata* | 90(5) | 117(13) | 200(52) | 2.68 | -0.694 | 0.189 | -0.860 |
| *A. bindusara* | 84(4) | 91(13) | 234(18) | 3.57 | -0.923 | 0.230 | -0.829 |
| *M. opalifer* | 148(6) | 48(5) | 418(38) | 7.44 | -1.93 | 0.570 | -0.577 |
| *C. atrata* | 85(5) | 90(8) | 462(34) | 6.13 | -1.59 | 0.236 | -0.825 |
|  | 95(5) | 90(8) | 410(49) | 5.55 | -1.44 | 0.264 | -0.804 |
